# Using quality indicators to predict inspection ratings: cross-sectional study of general practices in England

**DOI:** 10.3399/bjgp19X707141

**Published:** 2019-12-17

**Authors:** Thomas Allen, Kieran Walshe, Nathan Proudlove, Matt Sutton

**Affiliations:** Manchester Centre for Health Economics, Manchester.; Alliance Manchester Business School, Manchester.; Alliance Manchester Business School, Manchester.; University of Manchester, Manchester.

**Keywords:** data analysis, general practice administration and organisation, general practice standards, government regulation, National Health Service, patient safety

## Abstract

**Background:**

The Care Quality Commission regulates, inspects, and rates general practice providers in England. Inspections are costly and infrequent, and are supplemented by a system of routine quality indicators, measuring patient satisfaction and the management of chronic conditions. These indicators can be used to prioritise or target inspections.

**Aim:**

To determine whether this set of indicators can be used to predict the ratings awarded in subsequent inspections.

**Design and setting:**

This cross-sectional study was conducted using a dataset of 6860 general practice providers in England.

**Method:**

The indicators and first-inspection ratings were used to build ordered logistic regression models to predict inspection outcomes on the four-level rating system (‘outstanding’, ‘good’, ‘requires improvement’, and ‘inadequate’) for domain ratings and the ‘overall’ rating. Predictive accuracy was assessed using the percentage of correct predictions and a measure of agreement (weighted *κ*).

**Results:**

The model correctly predicted 79.7% of the ‘overall’ practice ratings. However, 78.8% of all practices were rated ‘good’ on ‘overall’, and the weighted *κ* measure of agreement was very low (0.097); as such, predictions were little more than chance. This lack of predictive power was also found for each of the individual domain ratings.

**Conclusion:**

The poor power of performance of these indicators to predict subsequent inspection ratings may call into question the validity and reliability of the indicators, inspection ratings, or both. A number of changes to the way data relating to performance indicators are collected and used are suggested to improve the predictive value of indicators. It is also recommended that assessments of predictive power be undertaken prospectively when sets of indicators are being designed and selected by regulators.

## INTRODUCTION

High-quality general practice is an essential component of a well-functioning healthcare system, but performance varies across practices.^1^^–^4 One approach that is increasingly used in an attempt to maintain and improve high standards of care is regulation,^5^ and most healthcare regulators across the world make use of some form of provider inspections.^6^ Typically, these are pre-announced visits that follow a set structure and evaluate providers against certain standards or requirements. Depending on the size of the provider and the intensity of the inspection, these visits can require significant amounts of time and human resources, and they are an expensive method of assessing quality. As a result of this high cost, it has been suggested that inspections should, where possible, be targeted where the potential for improvement is greatest and most needed.^7^

In England, health and social care providers are regulated by the Care Quality Commission (CQC), which was established in 2009 when the two previous regulators of health care and social care were merged. In the subsequent couple of years, several failures in care and management became very prominent in the media and politically. These included:

- excess deaths and poor care at Stafford Hospital, an acute hospital run by Mid-Staffordshire NHS Foundation Trust — in June 2010, the UK government announced there would be a public inquiry;
- patient abuse by staff at Winterbourne View private residential hospital for people with learning disabilities — this was exposed by a BBC television documentary in May 2011; and
- the July 2011 financial collapse of Southern Cross Healthcare Group, a private provider of health and social care services — at that time, it was the largest provider, with 31 000 residents in 750 care homes.^8^

These were noted in criticism of the CQC by the National Audit Office,^8^ the House of Commons Health Committee,^9^ and the Department of Health’s performance and capability review.^10^ It was also highlighted that between October 2010 and March 2011, the CQC had completed fewer than half of its planned number of inspections, because of prioritisation of registration over compliance and a shortage of inspectors.

The CQC responded by introducing a new, comprehensive inspection regime intended to help it *‘make better decisions about when, where, and what to inspect by using information and evidence in a more focused and open way’*.^11^ Part of this regime included developing a system, known as Intelligent Monitoring (IM), using performance indicators based on routinely collected and available data about providers. The IM system for general practices contained 33 performance indicators concerning patient satisfaction, the management of chronic conditions, prescribing, disease prevalence, and emergency hospital admissions. Together, these indicators were intended to support inspections with information on which practices to inspect and on which aspects of care to focus on.^12^^,^^13^ Performance indicators were aggregated into ratings of potential risk based on expected indicator values; these were to be used to create priority bands for inspection.^13^ Between September 2014 and January 2017, the CQC inspected 7330 practices, and awarded each of them one of four rating levels (‘outstanding’, ‘good’, ‘requires improvement’, or ‘inadequate’) in five domains (‘safe’, ‘effective’, ‘responsive’, ‘caring’, and ‘well led’), along with an ‘overall’ rating that summarised the domain ratings.

**Table:** How this fits in

Recent studies have questioned the utility of quality indicators in monitoring acute-hospital provider performance and targeting regulatory inspections; this study now extends this to general practice. It was found that the quality indicators in use from 2014 until 2017 were of little value in predicting subsequent inspection ratings and, as a result, suggestions regarding the future development of indicators include using more recent and up-to-date data, drawn from a wider range of sources, and considering changes over time.

Although the CQC’s IM indicators were not intended to replace inspections, this study examined whether they can be used to predict subsequent practice inspection ratings. This is important in terms of whether IM indicators can legitimately fulfil their aim to prioritise practice inspections or help determine the focus of inspection visits.

The equivalent IM system for secondary care has been found to be of limited value in prioritising hospital inspection or determining the focus of inspection visits.^14^^,^^15^ Recent research has examined the CQC inspection process and its impact on performance. Regarding the process, CQC inspectors have been found to disagree on judgements on vignettes of real inspection data, so assessments by teams are considered more reliable,^16^ and inspections were found to involve service users’ views, but in a transactional manner and on the CQC’s terms.^17^ Regarding their impact, IM indicators for acute hospital trusts have been found to lack predictive power^15^ and inspection of A&E departments has been found to have no impact on their performance metrics.^18^

## METHOD

### Data

The published inspection ratings and corresponding inspection dates for the 7330 GP practices that were inspected between September 2014 and January 2017 were obtained on request from the CQC. Over the inspection cycle, some practices were re-inspected and their ratings updated. Only the rating from the first inspection was used in this study, as subsequent ratings were likely to have been influenced by the previous inspection process and outcomes. Data on the most recent practice inspections are freely available online.^19^

For each of the inspected practices, the following data were obtained:

- the date of inspection;
- the date the rating was made public;
- the ‘overall’ rating; and
- the rating in each domain.

The IM dataset was also obtained from the CQC. There were two releases of IM, with indicator values being updated as new data were published. The second release^13^ was used; this covered the time period from April 2013 until December 2014 and, therefore, corresponded to the majority (90%) of inspections. There were no further updates to IM, so the indicator values for practices inspected later in the cycle were more than 2 years old.

The indicators were selected by the CQC through consultation and testing, and detailed definitions (Box 1) and data sources were made available.^13^ Data on one of the indicators — relating to emergency cancer admissions (GPHLIEC01) — were censored by the CQC for 1200 practices because of small numbers of admissions, resulting in a missing indicator value for these practices. As the statistical methods required a complete set of indicators, it was decided that this frequently missing indicator would be removed from the analysis. Each indicator was categorised by the CQC as relating to the ‘effective’, ‘caring’, or ‘responsive’ key domain; there were no indicators categorised to the other two domains (‘safe’ and ‘well led’) (Box 1). The raw indicator values were used to fully take account of the variations in indicators across practices.

**Box 1.** The 33 Intelligent Monitoring indicators for general practice

**Domain**	**Indicator code**	**Indicator description**
Effective	GPHLIAC01	Number of emergency admissions for 19 ambulatory care-sensitive conditions per 1000 population
Effective	GPHLIAP	Number of antibacterial prescription items prescribed per STAR PU
Effective	GPHLICH01	Ratio of reported versus expected prevalence for coronary heart disease
Effective	GPHLICPD	Ratio of reported versus expected prevalence for chronic obstructive pulmonary disease
Effective	GPHLICQI	Percentage of antibiotic items prescribed that are cephalosporins or quinolones
Effective	GPHLIEC01	Emergency cancer admissions per 100 patients on disease register
Effective	GPHLIFV01	Percentage of patients aged >6 months to <65 years in the defined influenza clinical risk groups that received the seasonal influenza vaccination
Effective	GPHLIFV02	Percentage of patients aged ≥65 years who have received a seasonal flu vaccination
Effective	GPHLIHP	Average daily quantity of hypnotics prescribed per STAR PU
Effective	GPHLIINI	Number of ibuprofen and naproxen items prescribed as a percentage of all non-steroidal anti-inflammatory drugs items prescribed
Responsive	GPPS001	Percentage of GP Patient Survey^a^ responders who gave a positive answer to the question *‘Generally, how easy is it to get through to someone at your GP surgery on the phone?’*
Caring	GPPS004	Percentage of GP Patient Survey responders who stated that they always or almost always see or speak to the GP they prefer
Caring	GPPS014	Percentage of GP Patient Survey responders who stated that the last time they saw or spoke to a GP, the GP was ‘good or very good’ at involving them in decisions about their care
Caring	GPPS015	Percentage of GP Patient Survey responders who stated that the last time they saw or spoke to a GP, the GP was ‘good or very good’ at treating them with care and concern.
Caring	GPPS020	Percentage of GP Patient Survey responders who stated that the last time they saw or spoke to a nurse, the nurse was ‘good or very good’ at involving them in decisions about their care
Caring	GPPS021	Percentage of GP Patient Survey responders who stated that the last time they saw or spoke to a nurse, the nurse was ‘good or very good’ at treating them with care and concern
Responsive	GPPS023	Percentage of GP Patient Survey responders who were ‘very satisfied’ or ‘fairly satisfied’ with their GP practice’s opening hours
Caring	GPPS025	Percentage of GP Patient Survey responders who described the overall experience of their GP surgery as ‘fairly good or very good’
Effective	QOFGP102	Percentage of patients with diabetes, on the register, in whom the last IFCC-HbA1c is ≤64 mmol/mol in the preceding 12 months
Effective	QOFGP104	Percentage of patients on the diabetes register, with a record of a foot examination and risk classification within the preceding 12 months
Effective	QOFGP106	Percentage of patients with diabetes, on the register, in whom the last blood pressure reading (measured in the preceding 12 months) is ≤140/80 mmHg
Effective	QOFGP110	Percentage of patients with schizophrenia, bipolar affective disorder, and other psychoses who had a comprehensive, agreed care plan documented in the record in the preceding 12 months
Effective	QOFGP111	Percentage of patients with schizophrenia, bipolar affective disorder, and other psychoses whose alcohol consumption has been recorded in the preceding 12 months
Effective	QOFGP150	Percentage of patients with atrial fibrillation (with CHADS^2^ score of 1), measured in the last 12 months, who are currently treated with anticoagulation drug therapy or an antiplatelet therapy
Effective	QOFGP155	Percentage of patients with hypertension in whom the last blood pressure reading measured in the preceding 9 months was ≤150/90 mmHg
Effective	QOFGP162	Percentage of patients with physical and/or mental health conditions whose notes recorded smoking status in the preceding 12 months
Effective	QOFGP178	Percentage of patients aged ≥75 years with a fragility fracture on or after 1 April 2012, who are currently treated with an appropriate bone-sparing agent
Effective	QOFGP182	Percentage of women aged 25–64 years whose notes record that a cervical screening test has been performed in the preceding 5 years
Effective	QOFGP27	Percentage of patients diagnosed with dementia whose care has been reviewed in a face-to-face review in the preceding 12 months
Effective	QOFGP33	Percentage of patients with diabetes, on the register, who have had a record of an albumin:creatinine ratio test in the preceding 12 months
Effective	QOFGP35	Percentage of patients with diabetes, on the register, whose last measured total cholesterol (measured in the preceding 12 months) was ≤5 mmol/l
Effective	QOFGP36	Percentage of patients with diabetes, on the register, who have had influenza immunisation in the preceding 1 September to 31 March
Effective	QOFGP55	Contractor has regular (at least every 3 months) multidisciplinary case review meetings, in which all patients on the palliative care register are discussed

a The GP Patient Survey is an independent survey run by Ipsos MORI on behalf of NHS England. The survey is sent out to over a million people across the UK. The results show how people feel about their GP practice. CHADS^2^ = Congestive heart failure, Hypertension, Age >75, Diabetes mellitus, and prior Stroke or transient ischaemic attack. IFCC-HbA1c = International Federation of Clinical Chemistry glycated haemoglobin (average plasma glucose concentration) measure. STAR PU = Specific Therapeutic group Age-sex Related Prescribing Unit.

The inspection ratings and IM datasets were merged to form one dataset containing rating and indicator data for the 6860 practices both monitored and inspected (470 practices were not included in the monitoring data).

### Statistical analysis

The rating levels were transformed into an ordered categorical variable ranging from 1 (‘inadequate’) to 4 (‘outstanding’). Ordered logistic regression was used to model the relationship between the ‘overall’ ratings and the 32 IM indicators. The same methods and modelling approach were applied to the three domain ratings on their domain-specific indicators. From these regression results, the probability that a practice received each of the four rating scores was predicted. These probabilities were multiplied by the rating score (1–4) and summed to arrive at a single, expected predicted value. This continuous predicted value was transformed to a discrete predicted rating, based on the following rules:

- predicted rating = 1 (‘inadequate’) if predicted values ≤1.5;
- predicted rating = 2 (‘requires improvement’) if predicted values were >1.5 and ≤ 2.5;
- predicted rating = 3 (‘good’) if predicted values were >2.5 and ≤ 3.5; and
- predicted rating = 4 (‘outstanding’) if predicted values were >3.5.

This approach and set of rules approximated a simple prediction model that the CQC could have used when prioritising inspections based on existing information.

After fitting the regression model and generating the predicted ratings, a measure of agreement (weighted *κ*) was used to compare the predicted with the actual ratings, in line with methodologies outlined by Cohen^20^ and Jakobsson and Westergren.^21^ Cohen’s weighted *κ* accounts for the level of agreement that would be expected by chance, and the test statistic measures the degree to which the predicted ratings improve — or not — on this level of chance agreement. Weighted *κ* ranges from −1 to 1, with a value close to 0 indicating little or no agreement beyond that expected through chance alone; a value close to 1 indicates strong agreement. A negative value indicates a level of agreement that is less than that expected by chance — that is, disagreement — while values in the 0.6–0.8 range indicate good agreement.^21^ The usual weighting system was applied, as follows:

- when predicted and observed ratings were equal, the weighting was 1;
- when ratings differed by one or two rating levels, the weighting was two-thirds, or one-third respectively; and
- when the difference was at the maximum (three rating levels), the weighting was 0.^20^^,^^21^

In an alternative approach, the four-level rating score was dichotomised by grouping ‘inadequate’ with ‘requires improvement’, and ‘good’ with ‘outstanding’. The probability of being in either of these two groups was estimated by logistic regression.

## RESULTS

**Figure 1.**
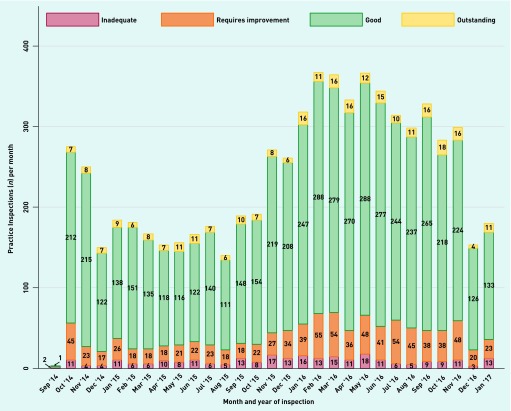
***Numbers of inspections, by month of inspection and split by ‘overall’ rating awarded in our dataset (*n *= 6860).^a^*** ***^a^The bars for Sep ‘14 depict two ‘inadequate’ inspections and one ‘good’ inspection.***

Figure 1 plots the inspections for each month dating from September 2014 until January 2017 for all 6860 practices in the dataset. The majority (5406 out of 6860, 78.8%) of practices were rated ‘good’; very few (275 out of 6860, 4.0%) were rated ‘inadequate’. A greater number of practices were inspected later in the cycle (from 2016 onwards) compared with earlier on. If the CQC had been using the IM dataset to prioritise inspections of practices that appeared to perform less well on the IM indicators, it could be expected that there would have been a pattern of more ‘inadequate’ and ‘requires improvement’ ratings earlier in the cycle; however, such a pattern was not apparent.

**Figure 2.**
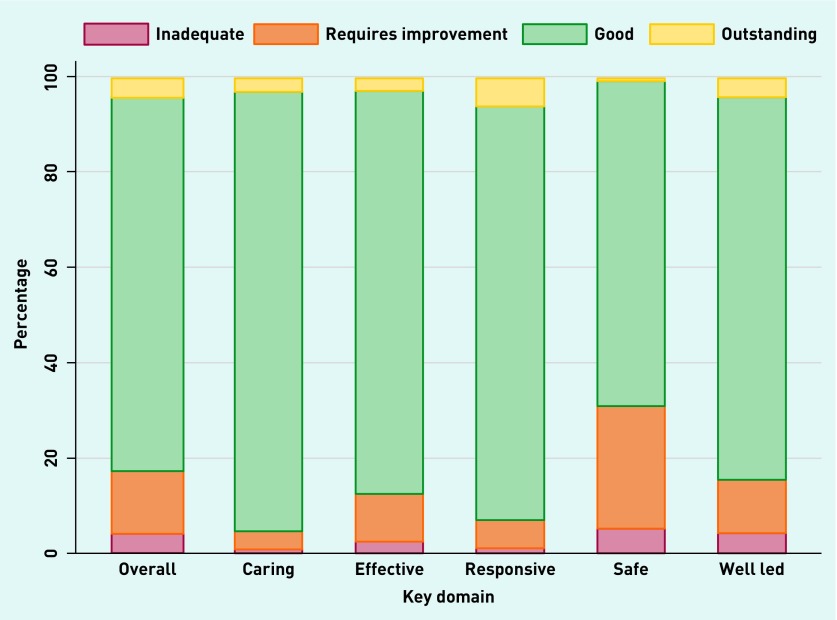
***Percentage of practices receiving each rating by key domain (*n *= 6860).***

Figure 2 plots the percentage of practices that received each rating for each of the five domains and for the ‘overall’ rating. The ratings were not particularly discriminating: in all domains, the majority of practices were rated ‘good’. The ‘caring’ domain had the highest proportion of ‘good’ ratings and the ‘safe’ domain had the lowest.

### Regression results

There were complete data on all 32 IM indicators for 5988 practices (87.3% of the dataset); these were used to predict the ‘overall’ rating. At the domain level, 6567 practices (95.7%) had complete data for the 24 indicators in the ‘effective’ domain, 6149 (89.6%) had complete data for all six indicators in the ‘caring’ domain, and 6835 (99.6%) for the two indicators in the ‘responsive’ domain.

Table 1 compares the predicted ‘overall’ ratings from the ordered logistic regression with actual ‘overall’ inspection ratings, and uses the percentage of correct predictions and weighted *κ* to assess the level of agreement. The regression model is shown in Table 2. It was found that 79.7% of actual ratings were predicted correctly; however, this occurred because the model predicted the great majority of practices to be rated ‘good’. Specifically, there were 4818 practices rated ‘good’ after inspection; 5756 practices had a predicted rating of ‘good’ and 4694 of these predictions were correct (81.5% accuracy) (Table 1).

**Figure 3.**
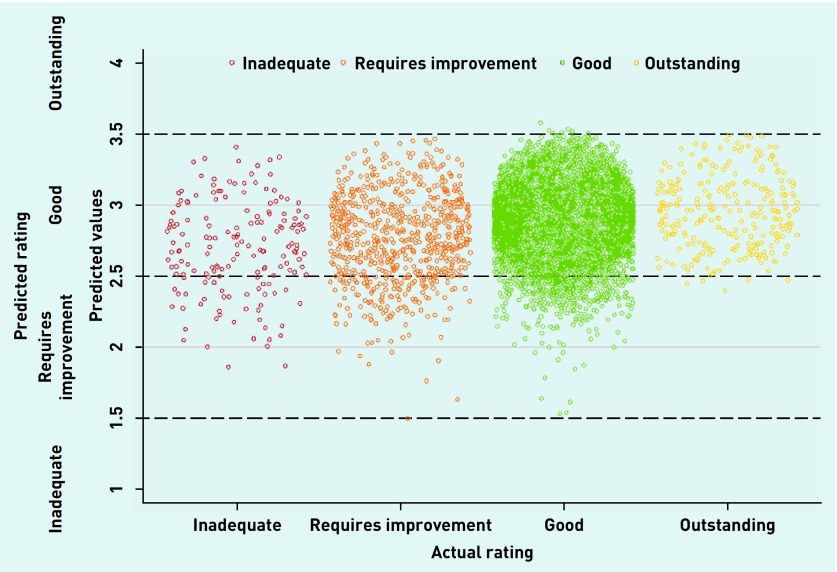
***Actual ‘overall’ ratings plotted against the predicted values from the ordered logistic regression model.^a,b^^a^Model shown in Table 2 (*n *= 5988). ^b^Data points were jittered to make the numbers of practices more apparent; horizontal, dashed lines indicate the cut points used to assign predicted ratings from predicted values.***

**Table 1.** Predicted and actual ratings from ordered logistic regression of ‘overall’ practice rating and all Intelligent Monitoring indicators^a^

**Actual practice rating**	**Predicted rating**				**Total**
	**Inadequate**	**Requires improvement**	**Good**	**Outstanding**	
Inadequate	**0**	32	140	0	172
Requires improvement	0	**76**	652	0	728
Good	1	123	**4694**	0	4818
Outstanding	0	0	270	**0**	270
Total	1	231	5756	0	5988
Percentage correct	79.7%				
weighted *κ*	0.097				

a Correct predictions are shown in bold. The full regression model is shown in Table 2.

**Table 2.** Ordered logistic regression of ‘overall’ practice rating on the 32 available Intelligent Monitoring indicators

**IM indicator**	**Odds ratio**	**95% CI**	***P*-value**
GPHLIAC01	1.013	0.997 to 1.028	0.104
GPHLIAP	0.283^a^	0.094 to 0.849	0.024
GPHLICH01	1.220	0.733 to 2.032	0.444
GPHLICPD	1.092	0.797 to 1.497	0.585
GPHLICQI	0.0861	0.006 to 1.298	0.076
GPHLIFV01	1.122	0.418 to 3.013	0.819
GPHLIFV02	6.206^a^	1.416 to 27.210	0.015
GPHLIHP	0.536^b^	0.353 to 0.814	0.003
GPHLIINI	5.881^c^	2.830 to 12.224	0.000
QOFGP102	7.373^c^	2.277 to 23.872	0.001
QOFGP104	1.625	0.581 to 4.550	0.355
QOFGP106	0.258^b^	0.093 to 0.712	0.009
QOFGP110	1.398	0.808 to 2.419	0.231
QOFGP111	1.041	0.466 to 2.325	0.921
QOFGP150	0.638	0.094 to 4.322	0.645
QOFGP155	4.139	0.830 to 20.646	0.083
QOFGP162	0.118	0.004 to 3.088	0.199
QOFGP178	0.548^b^	0.368 to 0.817	0.003
QOFGP182	2.100	0.610 to 7.229	0.239
QOFGP27	0.948	0.476 to 1.887	0.878
QOFGP33	1.226	0.465 to 3.233	0.681
QOFGP35	1.487	0.355 to 6.228	0.587
QOFGP36	2.349	0.754 to 7.319	0.141
QOFGP55	1.190^b^	1.066 to 1.328	0.002
GPPS004	0.273^c^	0.165 to 0.453	0.000
GPPS014	21.20^c^	3.906 to 115.055	0.000
GPPS015	7.722^a^	1.253 to 47.587	0.028
GPPS020	0.127^a^	0.023 to 0.696	0.017
GPPS021	2.253	0.327 to 15.544	0.410
GPPS025	8.077^b^	1.763 to 36.994	0.007
GPPS001	1.317	0.752 to 2.306	0.336
GPPS023	4.415^b^	1.535 to 12.694	0.006
Observations	5988		
Pseudo *R*^2^	0.071		

a P*< 0.05.*

b P*< 0.01.*

c P*< 0.001. CI = confidence interval. IM = Intelligent Monitoring.*

The prediction model performed most poorly at identifying the practices most in need of inspection, that is, those that received a rating of ‘inadequate’ or ‘requires improvement’. There were 172 practices with an ‘inadequate’ inspection rating and none were predicted correctly (0.0% accuracy). Likewise, 728 practices had an inspection rating of ‘requires improvement’, but only 76 of these were predicted correctly (10.4% accuracy). The model also predicted that none of the 270 practices that were deemed ‘outstanding’ would be awarded such a rating (0.0% accuracy).

The weighted *κ* statistic of 0.097 meant that predicted ratings were 9.7% of the way between the level of agreement expected by chance and perfect agreement — this is very low, and far below the 0.6–0.8 range that would be considered good agreement.^20^^,^^21^

Figure 3 illustrates this point graphically by plotting the predicted inspection rating scores (prior to rounding to discrete categories) against actual inspection ratings. There was substantial overlap between the ranges of predicted inspection ratings for each of the four sets of actual inspection rating outcomes. Comparable models and predictions for the ‘caring’, ‘effective’, and ‘responsive’ domains are not shown but performed worse, with fewer accurate predictions and lower weighted *κ* statistics.

The dichotomised ratings score was used in a binary logistic regression to predict the probability of receiving a poor inspection rating (‘inadequate’ or ‘requires improvement’) compared with a good inspection rating (‘good’ or ‘outstanding’), based on IM indicator values. The predicted probabilities for both groups indicated a higher degree of overlap than shown in Figure 3 and it was not possible to distinguish practices in greater need of inspection.

## DISCUSSION

### Summary

The performance indicators in the CQC’s IM dataset were found to have very limited ability to predict the inspection ratings of general practices in advance of inspection, and they also could not separate practices as being merely good or bad. Moreover, predictions were more likely to underestimate the need for inspection by predicting practices to have a better rating than was found on inspection. A data-driven approach to the prioritisation of general practice inspections using the IM indicators is unlikely to be effective unless significant improvements are made.

### Strengths and limitations

This study used all but one of the IM indicators (emergency cancer admissions), and their raw values, examination of all domains with allocated indicators, and the large dataset of GP practices. Had the models found a reasonable fit to this entire dataset, split-sample testing or cross-validation could have been conducted to get a fairer estimation of the predictive accuracy by fitting a model to a subset of GP practices chosen randomly or by inspection date (temporal validation),^22^ and then applying the model to obtain predictions for the remainder of practices. The predictive ability of such out-of-sample testing would likely be lower than that found in this study’s whole-dataset results because the model would have been tested on a subset of data different from that to which it had been fitted (tuned).

One limitation was inherent in the inspection dataset: as actual inspection ratings were so undiscriminating, and were asymmetrically distributed across the four categories with nearly 80% of ‘overall’ ratings being ‘good’, it would have been difficult for any model to achieve predictive accuracy better than chance. Furthermore, it was only possible to test the power of IM at predicting domain ratings when the IM indicators were targeted at those domains (‘effective’, ‘caring’, and ‘responsive’). It was not possible to do this for the domains of ‘well led’ and ‘safe’, as these had no IM indicators; practices tended to have poorer ratings in these domains.

### Comparison with existing literature

A similar lack of predictive ability was found when analysing the IM system for acute hospitals, both overall,^14^ and on the underlying domains (domains such as ‘safe’ and ‘caring’).^15^ There have not been any studies that find value in the IM data.

### Implications for research and practice

It should be noted that CQC guidance on IM says that it was used to *‘raise questions, not make judgements’*,^13^ and that inspections draw on a much wider range of both quantitative and qualitative data; as such, one would not necessarily expect to find a very high level of agreement between predicted and actual inspection ratings. However, as so little agreement was found, it has brought into question how useful the IM indicators are to the inspection programme — it could also throw doubt on the validity and reliability of CQC inspection ratings, the IM indicators, or both, as measures of practice quality. The findings presented here raise important questions about what those indicators and the CQC inspection ratings were supposed to be measuring. If both sought to measure the quality of care or wider aspects of general practice performance, it is perhaps a surprise and a cause for concern that there seems to be so little agreement between them. Of course, the fact that CQC ratings for general practices were so unevenly distributed made it statistically more difficult for any dataset to have predictive value.

In its strategy for 2016–2021, the CQC has articulated its aim to move to a more intelligence-led model of regulation and inspection across all health and social care sectors, along with its plans to replace IM with a new system named CQC Insight.^23^ Given that, it is worth considering the lessons that can be drawn from the research presented here. It is always important to be clear about the purpose of routine monitoring of performance indicators, and to incorporate testing and validation into their development and piloting before they are deployed in routine use. It may be attractive to make use of existing and available datasets, but their validity as measures of the quality of care or wider performance should be explored and tested. Such datasets will never be capable of predicting the outcomes of inspection perfectly (and, of course, if they were, there would be no need for such inspections), because inspections are able to draw on a much wider range of qualitative and quantitative data, as well as on the expertise and judgement of the inspection team. However, one should expect IM, CQC Insight, and other forms of routine monitoring to have some predictive value. It may be possible to improve their predictive value by using:

- more recent and up-to-date data;
- time-series data to take into account changes in provider performance over time; and
- a wider range of data from other sources, such as patient feedback.^24^

In conclusion, it seems that CQC and other regulators should test and evaluate the validity and reliability of both performance indicator systems and inspection rating schemes if and when they are introduced; and arguably should continue to monitor validity and reliability routinely. If their predictive value cannot be demonstrated, this will limit the scope for CQC and other regulators to use risk-based or responsive regulatory models that target regulatory interventions such as provider inspections based on their reported performance.
